# Left supraclavicular (Virchow’s) node metastasis detected before primary infradiaphragmatic tumor: a case series

**DOI:** 10.1186/s13256-022-03261-6

**Published:** 2022-01-26

**Authors:** Yumi Mochizuki, Maiko Tsuchiya, Jun Oyama, Akane Wada, Takuma Kugimoto, Takeshi Kuroshima, Hideaki Hirai, Hirofumi Tomioka, Hiroyuki Harada, Tohru Ikeda, Takumi Akashi

**Affiliations:** 1 Department of Oral and Maxillofacial Surgery, Graduate School, Tokyo Medical and Dental University, 1-5-45, Yushima, Bunkyo-ku, Tokyo, 113-8549 Japan; 2 Department of Oral Pathology, Graduate School, Tokyo Medical and Dental University, Tokyo, Japan; 3 Department of Nuclear Medicine, Graduate School, Tokyo Medical and Dental University, Tokyo, Japan; 4 Division of Diagnostic Radiology, Graduate School, Tokyo Medical and Dental University, Tokyo, Japan

**Keywords:** Intrahepatic cholangiocarcinoma, Bladder carcinoma, Prostate carcinoma, Left supraclavicular (Virchow’s) node, Left supraclavicular node metastasis, Oral cancer

## Abstract

**Background:**

Metastasis of infradiaphragmatic tumors to the left supraclavicular lymph node is reported to be rare. When metastasis is detected in the left supraclavicular node in patients with head and neck carcinoma, locating the primary cancer remains a difficult and time-consuming challenge despite the dramatic development of screening technologies and treatment methods.

**Case presentation:**

We report three cases of malignant infradiaphragmatic tumor diagnosed following an initial finding of left supraclavicular node metastasis after surgery for tongue squamous cell carcinoma (follow-up period, range 18–62 months). In these cases, adenocarcinoma was diagnosed based on left supraclavicular node biopsies, and a second primary tumor was found, in a 78-year-old Japanese woman with a diagnosis of cholangiocarcinoma, a 64-year-old Japanese man with a diagnosis of bladder carcinoma, and a 61-year-old Japanese man with a diagnosis of prostate carcinoma. In the cholangiocarcinoma case, carbohydrate antigen 19-9 and alpha-fetoprotein levels helped to diagnose cholangiocarcinoma. Palliative care only was given, with survival for 11 months after diagnosis of lymph node metastasis. In the bladder carcinoma case, pathological analysis of fine-needle aspiration biopsy specimen of the metastatic cervical lymph node showed atypical cells with slight squamous differentiation. Hematoxylin–eosin staining of the bladder lesion did not identify a clear glandular or squamous component, and we could not make a definitive diagnosis of whether the lesion was poorly differentiated squamous cell carcinoma, adenocarcinoma, or high-grade urothelial carcinoma. GATA3 staining aided in the diagnosis of urothelial bladder cancer with left supraclavicular node metastasis. He survived for 2 months after diagnosis of left supraclavicular lymph node metastasis. In the prostate carcinoma case, ^18^F‐fluorodeoxyglucose uptake was weak. Prostate-specific antigen levels and magnetic resonance imaging findings aided the diagnostic process. This patient underwent bilateral orchiectomy and adjuvant hormonal therapy and survived for 47 months after diagnosis of left supraclavicular node metastasis.

**Conclusions:**

Pathological diagnosis on the basis of immunohistochemistry and specific diagnosis methods such as radiological and serological assessments are important for providing rapid diagnosis and appropriate treatment.

## Introduction

Since Virchow's time, the survival rates of cancer patients have improved because of dramatic research advances in the epidemiology of cancer and the development of screening technologies and treatment methods.

Supraclavicular lymph node metastasis has been reported to be more common in patients with lung and breast tumors (18.7% and 18.6%, respectively) [1, 2]. However, when metastasis is detected in the left supraclavicular node in patients with head and neck carcinoma, locating the primary cancer remains a difficult and time-consuming challenge. The accumulation of cases and the search for optimal therapeutic strategies remain important [2].

Patients with oral squamous cell carcinoma and those with other types of head and neck cancer are at higher risk of second primary cancer than patients with cancers at most other sites [3]. Mroueh *et al*. found a second primary cancer in 10% of patients with oral squamous cell carcinoma in their cohort [3], while Min *et al*. reported that the risk of a second primary oral cancer was higher (standardized incidence ratio 16.25, 95% confidence interval 13.04–20.02) than that of nonoral second primary cancer (standardized incidence ratio 1.37, 95% confidence interval 1.29–1.45) in Korean patients with cancer in the oral cavity [4]. In patients with tongue carcinoma, the second primary cancer most frequently occurs in the oropharynx, followed by the esophagus and larynx [3, 4]. In contrast, second primary cancer at other sites, such as the intrahepatic bile duct, bladder, and prostate, is rare in patients with tongue carcinoma.

Metastasis of infradiaphragmatic tumors to the left supraclavicular lymph node is also reported to be rare. The incidence of metastasis has been reported to be 3.6% for intrahepatic cholangiocarcinoma [1], 1.4% for bladder carcinoma [5], and approximately 0.3% for prostate carcinoma [6–8].

We encountered three patients with initial findings of left supraclavicular lymph node metastasis before detection of the primary infradiaphragmatic tumor during follow-up after surgery for tongue cancer. Here, we report these three cases and describe our diagnostic strategy.

## Case series

### Case 1

**Fig. 1:**
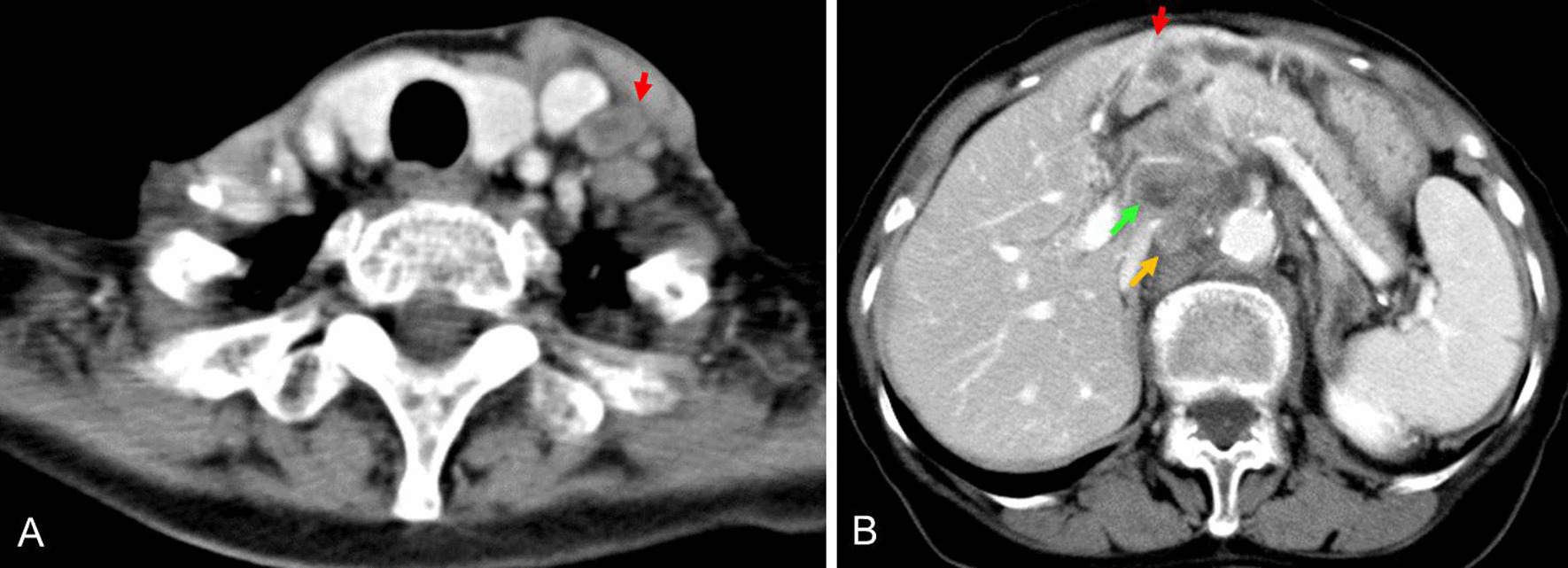
Enhanced computed tomography scans. **A** Enhanced computed tomography scan of left supraclavicular lymph node (red arrow). **B** Abdominal enhanced computed tomography scans. An ill-defined lesion with a low-density computed tomography value was observed in the intrahepatic bile duct (red arrow). Multiple paraaortic lymph nodes (yellow arrow) and periportal lymph nodes (green arrow) were swollen

**Fig. 2:**
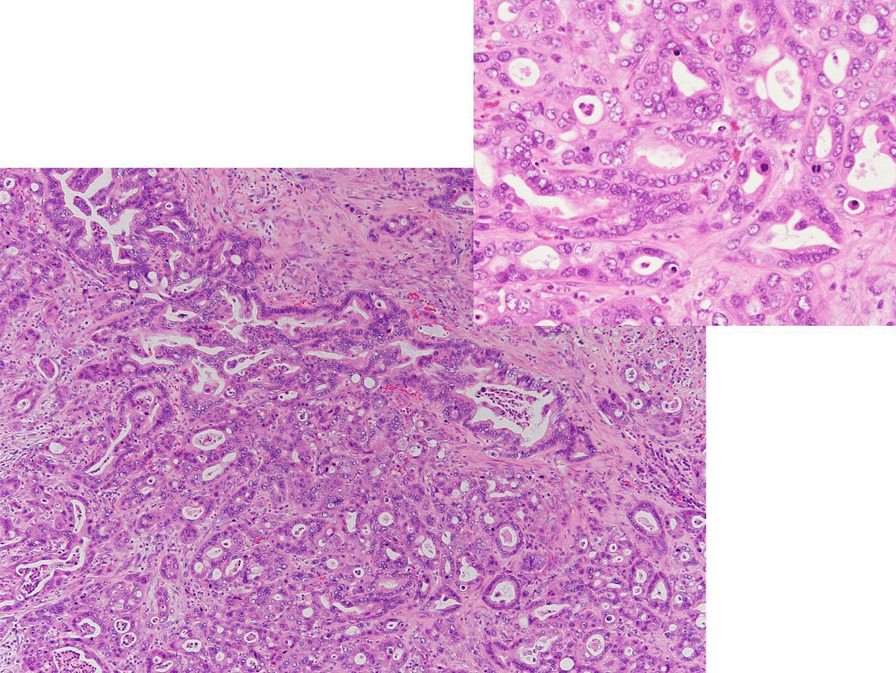
Pathological image of H–E staining of the left supraclavicular lymph node. The diagnosis was adenocarcinoma (left side of the photograph, ×100). Tumor cells constructed well-developed glands (right side of the photograph, ×400)

A 78-year-old Japanese woman with a diagnosis of tongue carcinoma underwent partial glossectomy in May 2002 and ipsilateral neck dissection (levels I–V) in July 2002. The pathological diagnosis was poorly differentiated squamous cell carcinoma (pT1N2bM0, according to the Union for International Cancer Control (UICC) TNM classification, 7th edition). She had no notable medical history at the time of her treatment for tongue carcinoma. We subsequently palpated left-sided supraclavicular lymphadenopathy at a postsurgical follow-up visit in July 2003 (Fig. 1A). Biopsy of the left supraclavicular lymph node yielded a diagnosis of adenocarcinoma (Fig. 2). Her carbohydrate antigen 19-9 (CA19-9) and alpha-fetoprotein levels were elevated to 4100 U/mL and 49.0 ng/dL, respectively. Infiltrative intrahepatic cholangiocarcinoma was detected on whole-body computed tomography (CT) scan (Fig. 1B). The patient consented to additional tissue biopsy of the bile duct, and the clinical diagnosis was cholangiocarcinoma (T2N1M1). She opted for palliative care and died 11 months after detection of left supraclavicular lymph node metastasis.

### Case 2

**Fig. 3:**
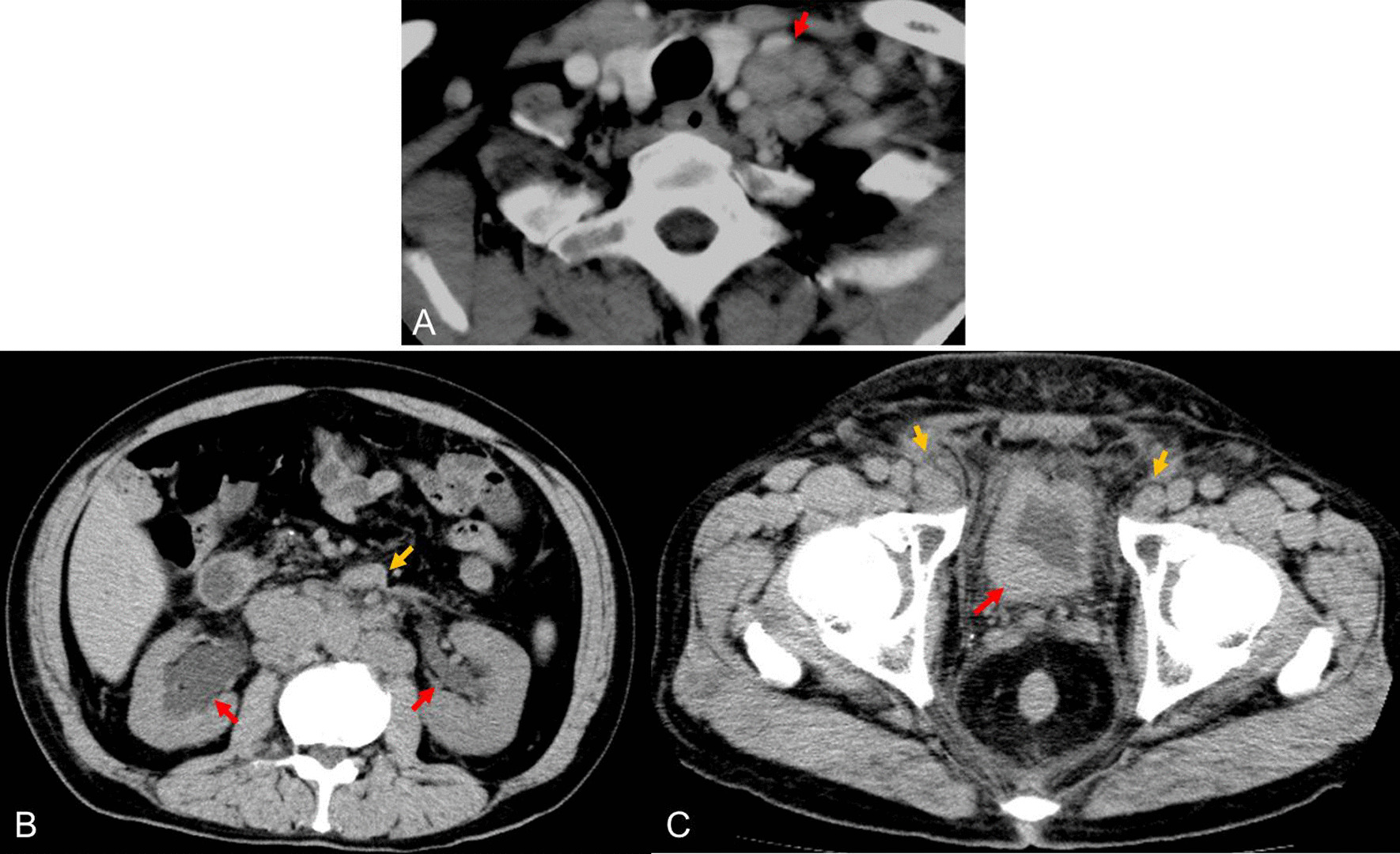
Enhanced computed tomography scans. **A** Enhanced computed tomography scan of left supraclavicular lymph node (red arrow). **B** Abdominal enhanced computed tomography scans. Bilateral hydronephrosis (red arrow) and paraaortic lymphadenopathy (yellow arrows) were observed. Pelvic enhanced computed tomography scans. **C** An ill-defined lesion in the bladder was observed (red arrow), and bilateral inguinal lymphadenopathy was observed (yellow arrow)

**Fig. 4:**
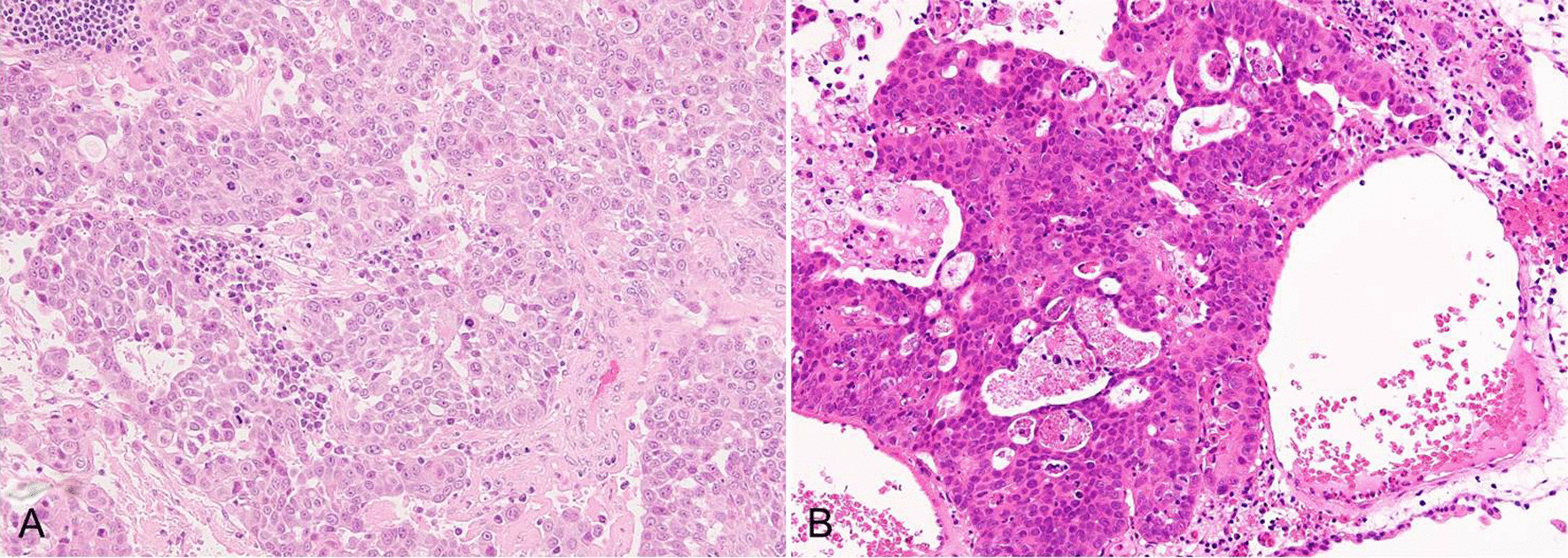
Pathological image of H–E staining. **A** Pathological image of the left supraclavicular lymph node (×100). Tumor cells were organized into irregular nests. Squamous differentiation was found in some sections. **B** Pathological image of the bladder lesion (× 200). Visualization of the ductal components and keratinization was not clear

**Fig. 5:**
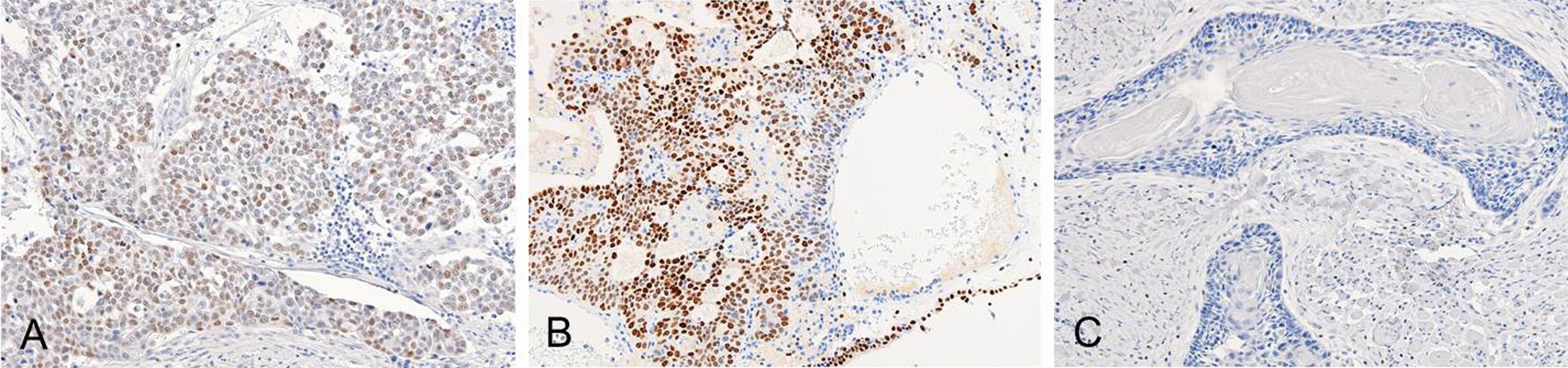
Pathological image of GATA3 staining. **A** Pathological image of the left supraclavicular lymph node (×200). **B** Tumor cells were positive for GATA3. Pathological image of the bladder lesion (×200). **C** Tumor cells were positive for GATA3. Pathological image of the tongue lesion (×200). Tumor cells were negative for GATA3

A 64-year-old Japanese man underwent ipsilateral neck dissection (levels I–III), partial glossectomy, and forearm flap reconstruction for moderately differentiated squamous cell carcinoma of the tongue in February 2007. His medical history at the time when tongue carcinoma was detected included chronic otitis media. The pathological diagnosis was poorly differentiated squamous cell carcinoma (pT2N1M0). He revisited our department in March 2012 after noticing left supraclavicular lymphadenopathy (Fig. 3A). Fine-needle aspiration biopsy of the left supraclavicular lymph node revealed atypical cells with slight squamous differentiation, which was diagnosed as class IV, and biopsy of the left supraclavicular lymph node led to a diagnosis of carcinoma (Fig. 4A). Primary cancer screening was started in the head and neck and lung regions, but during that time, he developed postrenal renal failure (Fig. 3B), and a bladder tumor was found on an enhanced pelvic CT scan (Fig. 3C). Hematoxylin–eosin staining of the bladder lesion did not identify a clear glandular or squamous component, and we could not make a definitive diagnosis of whether the lesion was poorly differentiated squamous cell carcinoma, adenocarcinoma, or high-grade urothelial carcinoma (Fig. 4B). Immunopathological examination of the left supraclavicular lymph node showed CK7(+), CK20(−), and GATA3(+) (Fig. 5A), and the bladder lesion showed CK7(+), CK20(−), and GATA3(+) (Fig. 5B). On the other hand, immunopathological examination of the squamous cell carcinoma on the tongue showed GATA3(−) (Fig. 5C). Based on these pathological findings, the diagnosis was compatible with primary bladder cancer with metastasis to the left supraclavicular lymph node (pT2N2M1). The patient opted for palliative care and died 2 months later.

### Case 3

**Fig. 6:**
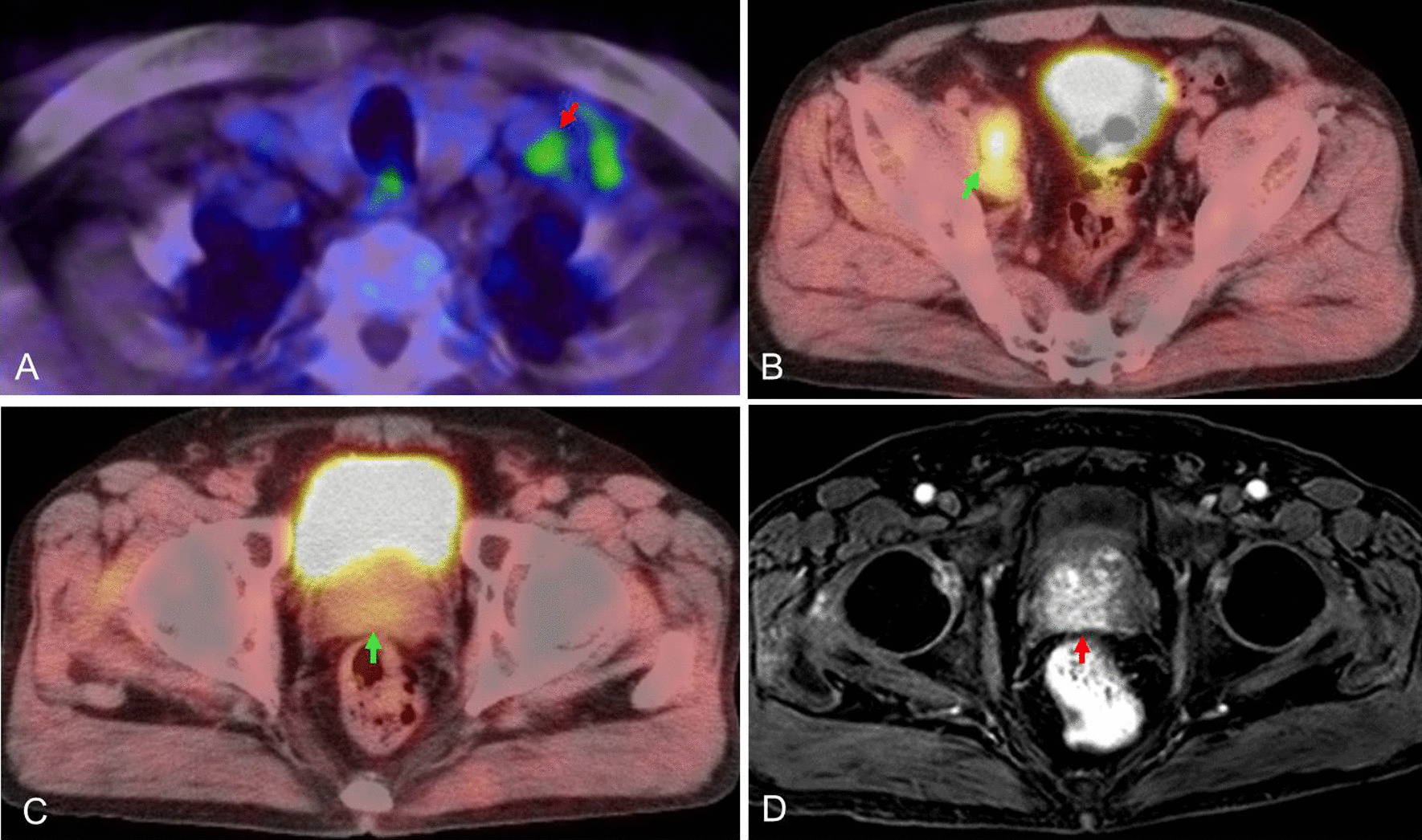
^18^F-FDG PET/CT and MRI findings. **A** ^18^F-FDG PET/CT of the cervical lesion. ^18^F-FDG uptake was observed in the left supraclavicular lymph node (red arrow). **B** ^18^F-FDG PET/CT of the pelvic lesion (green arrow). Strong accumulation of ^18^F-FDG in the right obturator lymph node was detected. **C** ^18^F-FDG PET/CT of the pelvic lesion (green arrow). Weak ^18^F-FDG uptake was observed in the prostate (green arrow). **D** A contrast-enhanced MRI scan of the pelvis (fat-saturated contrast-enhanced T1-weighted image). An enhanced lesion was seen in the prostate (red arrow)

**Fig. 7:**
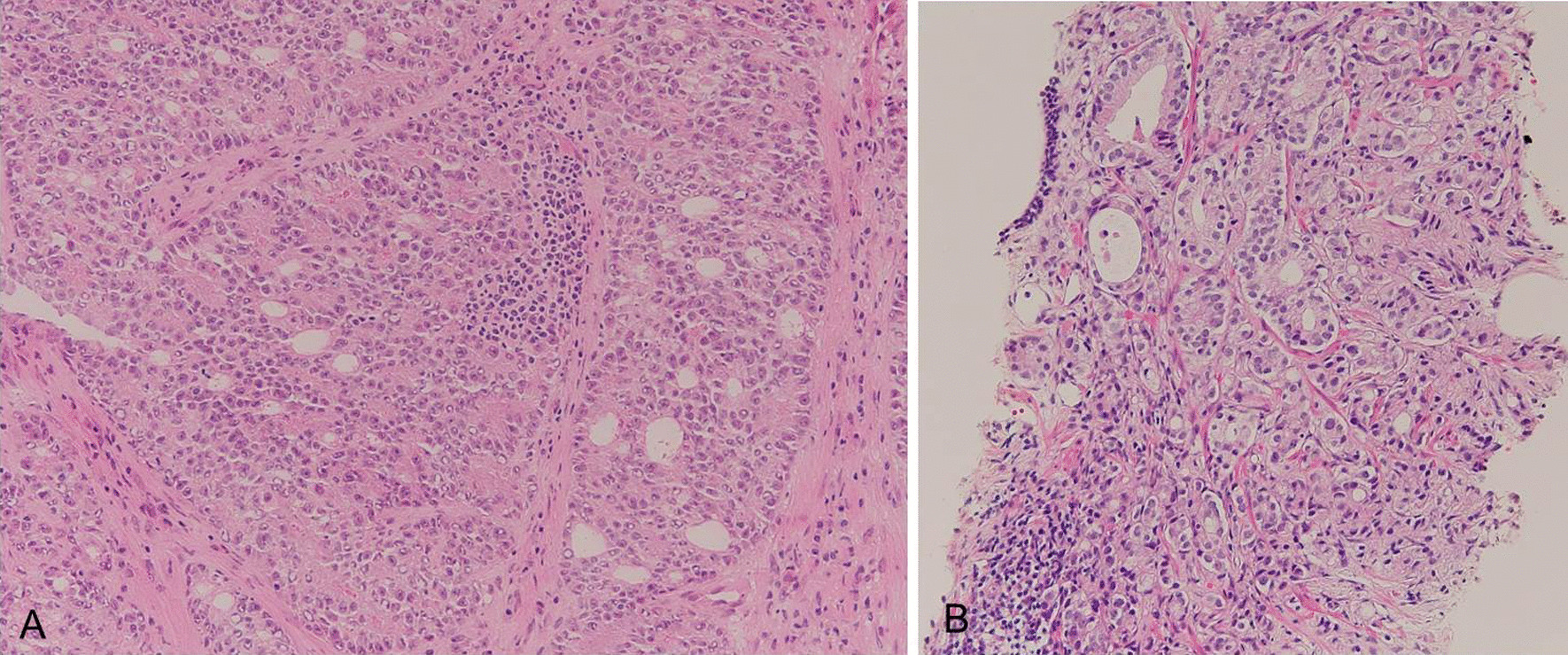
Pathological image of H–E staining. **A** Pathological image of the left supraclavicular lymph node (×200). Cribriform glands were recognized. The diagnosis was adenocarcinoma. **B** Pathological image of the prostate lesion (×200). Mixed solid sheet-like growth and cribriform glands were observed. The diagnosis was adenocarcinoma of the prostate lesion

A 61-year-old Japanese man underwent ipsilateral neck dissection (levels I–III), partial glossectomy, and forearm flap reconstruction for poorly differentiated squamous cell carcinoma on the right side of the tongue in March 2011. His medical history included hypertension and a duodenal ulcer. The pathological diagnosis was poorly differentiated squamous cell carcinoma (pT2N0M0). Follow-up ^18^F-fluorodeoxyglucose (FDG) positron emission tomography/computed tomography (^18^F‐FDG‐PET/CT) in March 2013 showed intense ^18^F‐FDG accumulation in the left supraclavicular (Fig. 6A) and right obturator (Fig. 6B) lymph nodes. ^18^F‐FDG uptake in the prostate was weak (Fig. 6C). Biopsy of the left supraclavicular lymph node gave a diagnosis of adenocarcinoma (Fig. 7A). Contrast-enhanced magnetic resonance imaging (MRI) of the pelvis showed adenopathy of the obturator nodes on the right side of the prostate region and an enhancing lesion in the prostate on contrast-enhanced T1-weighted images (Fig. 6D). Biopsy of the prostate revealed adenocarcinoma with Gleason score of 9 (4 + 5) that was diagnosed as prostate carcinoma (pT2N1M1) (Fig. 7B). His prostate-specific antigen (PSA) level at time of diagnosis was elevated at 123.9 ng/mL. Bilateral orchiectomy and adjuvant hormonal therapy were performed. He died 47 months after diagnosis of left supraclavicular lymph node metastasis.

## Discussion

Metastasis from an infradiaphragmatic tumor to the left supraclavicular lymph node occurs via the rich lymphatic network of the retroperitoneal lymph nodes, cisternae chyli, and the thoracic duct, which drains into the systemic circulation via the left subclavian vein. Infradiaphragmatic tumor metastases to the lymph nodes of the head and neck without lung involvement are considered to occur via the vertebral venous plexus system [9].

Morphologically, the vertebral venous plexus system shows interindividual variability with little or no valves and many branches [10]. Therefore, tumor nests encounter little resistance as they ascend through the vertebral venous plexus when the intraabdominal or intrathoracic pressure is increased [10], and these tumor nests do not pass through the lung [11].

### Diagnosis

Combined radiological and serological assessments are useful for the diagnosis of primary cancer. CA19-9 and alpha-fetoprotein levels helped make the diagnosis in our first case, as did the PSA level in the second case. ^18^F‐FDG‐PET/CT has greatly improved our ability to detect an unknown primary tumor, particularly at a distant location. However, ^18^F-FDG PET/CT sometimes fails to reveal organ-localized prostate cancer [12–15]. In our third case, the ^18^F‐FDG uptake was weak on ^18^F-FDG-PET/CT and MRI findings aided the diagnosis. Thus, prostate cancer cannot be ruled out even if the uptake is weak, and careful examinations, such as PSA measurements, should be considered.

### Pathology

In our second case, because of the presence of atypical cells with slight squamous differentiation in the fine-needle aspiration biopsy and the lymph node specimen, the difficulty of obtaining a definitive pathological diagnosis from excisional biopsy of the left supraclavicular lymph node, and the patient’s history of squamous cell carcinoma of the tongue, primary cancer screening was started in the head and neck and lung regions. However, the lymph node metastasis originated from the bladder. The histological presentation of urothelial carcinoma is very similar to that of squamous cell carcinoma [11, 16, 17]. If histopathological examination reveals squamous differentiation in metastatic cervical lymph nodes, it is necessary to include the bladder in the search for the primary cancer site [16, 18]. In this case, GATA3(+) staining of the metastasis to the supraclavicular lymph node revealed that the primary cancer was not squamous cell carcinoma of the tongue but urothelial carcinoma in the bladder. The CK7(+) and CK20(−) phenotypes indicate a high probability of bladder urothelial carcinoma [19]. Combined immunolabeling for CK7 and CK20 often aids in the identification of urothelial bladder cancer; however, these markers have limited sensitivity and specificity [19]. Immunohistochemistry of the transcription factor GATA3, a sensitive and specific diagnostic urinary epithelial marker, has been widely used in the diagnosis of urothelial cancer [19, 20].

### Prognosis

Our first patient opted for palliative care only and survived for 11 months after diagnosis of lymph node metastasis. There have been some reports of improved survival (for more than 6 years) after successful chemotherapy treatment for metastasis of cholangiocarcinoma to Virchow’s node [21, 22]. Therefore, the prognosis of patients with intrahepatic cholangiocarcinoma and metastasis to the left supraclavicular lymph node is not always poor [21].

The prognosis has been consistently poor in the limited number of case reports on cervical lymph node metastasis from bladder carcinoma [23, 24]. The median survival of patients with metastatic bladder cancer who receive supportive care alone is 4–6 months [18]. Our second patient, who chose palliative care, survived for only 2 months after diagnosis of left supraclavicular lymph node metastasis. As in previous reports, his prognosis was poor.

Even for patients with advanced-stage prostate adenocarcinoma and metastasis to the head and neck region, survival may be extended by rapid diagnosis and appropriate treatment [25]. Hormonal therapy has been shown to prolong survival, even for patients with metastasis of prostate adenocarcinoma to Virchow’s node [26]. One study found that the average survival time was 25.8 (range 1–101) months for patients with prostate adenocarcinoma and Virchow’s node metastasis and pointed out that the prognosis of these patients was superior to that of patients with metastatic adenocarcinoma of nonprostatic origin [25]. Our third patient survived for 47 months after diagnosis of left supraclavicular lymph node metastasis.

The prognosis of malignant infradiaphragmatic tumors after metastasis to Virchow’s node is generally considered to be extremely poor [21]. However, our experience suggests that the prognosis may depend on the characteristics of the tumor and that rapid diagnosis is important for providing appropriate treatment. Enlarged left supraclavicular lymph nodes can be more easily palpated and visually inspected than nodes in other areas of the body. If enlarged left supraclavicular lymph nodes are detected on routine clinical examination, clinicians should request additional assessments such as CT, MRI, ultrasonography, and PET/CT to ensure rapid detection and diagnosis of the primary lesion. In addition, biopsy of the enlarged cervical lymph nodes and serological assessments should be conducted to diagnose the primary cancer.

Further accumulation of cases and more detailed studies of malignant infradiaphragmatic tumors with Virchow’s node metastasis are needed.

## Conclusion

We encountered three patients with malignant infradiaphragmatic tumors in whom the initial diagnosis was left supraclavicular lymph node metastasis. Pathological diagnosis and specific diagnosis methods such as radiological and serological assessments are important for providing rapid diagnosis and appropriate treatment. Survival time varied according to the characteristics of the specific tumor type.

## Data Availability

The datasets used and/or analyzed during the current study are available from the corresponding author on reasonable request.

## Abbreviations

CA19-9: Carbohydrate antigen 19-9
CT: Computed tomography
PSA: Prostate-specific antigen
^18^F-FDG PET/CT: ^18^F-fluorodeoxyglucose positron emission tomography/computed tomography
